# A Practical Treatise on Fractures and Dislocations

**Published:** 1860-07

**Authors:** 


### NORTH AMERICAN

### MEDICO-CHIRURGICAL REVIEW.

JULY, 1860.

### Analytical and Critical Reviews.

ART. I.—*A Practical Treatise on Fractures and Dislocations.* By FRANK HASTINGS HAMILTON, M.D., Professor of Surgery in the University of Buffalo, Surgeon to the Buffalo Hospital of the Sisters of Charity, etc. etc. Illustrated with Two Hundred and Eighty-nine Wood-cuts. 8vo., pp. 757. Blanchard & Lea: Philadelphia, 1860.

THE medical literature of our country will be proud to hail the appearance of this work as a contribution of no ordinary value. The able, learned, and industrious author has performed his task with great fidelity, and his professional brethren everywhere will gladly accord to him the highest praise for having placed at their command a treatise to which they may constantly refer, in the hour of doubt and perplexity, as a safe and ready guide. From the great labor and time bestowed upon its preparation, we had been led to anticipate a very thorough and elaborate monograph, and an attentive perusal of its pages has satisfied us that our expectations have been fully realized. The work is by far the most complete disquisition on fractures and dislocations in the English language, and, with the exception of that of M. Malgaigne, indeed the most complete in any language. We are rejoiced that this task has been reserved for an American surgeon, and doubly rejoiced that the task has been so well and so creditably executed.

Until the appearance of the present treatise our literature was sadly deficient upon the subject of fractures and dislocations. The work of Boyer, edited by Richerand, translated by Farrell, and reissued in this country early in the present century, under the supervision of Dr. Hartshorne, of this city, had long been obsolete, notwithstanding its great merit as a full and systematic monograph at the time of its publication.

The admirable treatise on dislocations and on fractures of the joints, by Sir Astley Cooper, was never intended to cover the entire surface of those important branches of pathology and practice; and as to the productions of Amesbury and Lonsdale, they certainly had no claim to any exalted position in medical literature, that of the former being designed chiefly to illustrate some peculiar methods of practice, while that of the latter was anything but full or circumstantial in its details. The German language possessed the work of A. L. Richter, entitled "Theoretisches Praktisches Handbuch der Lehre von den Brüchen und Verrenkungen der Knochen," issued at Berlin in 1828, and accompanied by a folio volume of illustrations; but in consequence of our ignorance of that language, the treatise found but few readers on this side of the Atlantic. The treatise of Mr. Robert M. Smith, of Dublin, on fractures in the vicinity of the joints, and on certain forms of accidental and congenital dislocations, was a most valuable monograph, replete with original observations and practical suggestions; but it failed to supply the great void which had so long existed in our literature upon the subject under review. It was not until a few years ago that an attempt was made to fill this gap in a manner at all commensurate with the wants of the age, or the actual demands of science and humanity. Malgaigne, in 1847, published the first volume of his great work on fractures and dislocations, the second being issued eight years afterwards, the whole comprising nearly two thousand pages, with a folio atlas of plates. Of this treatise, which impartial criticism must regard as one of the most valuable contributions to surgical science in the nineteenth century, the portion relating to fractures was ably translated by Dr. Packard, of this city, and published by Messrs. Lippincott & Co. early in 1859. Whether the other portion will meet with a similar favor is somewhat doubtful, especially now that the work of Dr. Hamilton has supplied the want that had previously existed upon the subject. Very recently a treatise on fractures appeared in Germany, from the pen of Dr. Gurlt, comprising a more complete view of the subject than any other work that has yet been issued in that language. We must not omit here to mention also the splendid volume on "Dislocations and Fractures," lately published by Mr. Joseph Maclise, of London, an imperial folio, accompanied by numerous plates illustrative of the various forms of these accidents, and executed in the highest style of the art. The remarks upon anatomy, pathology, etiology, and treatment of the lesions delineated in the plates are, throughout, characterized by good sense; but the work, nevertheless, fails to come up to our ideas of what such a treatise ought to be at the middle of the nineteenth century. As a book of reference, in cases of sudden emergencies, it is invaluable.

From the foregoing remarks, it will be perceived that the work of Professor Hamilton has appeared at a most opportune period, and it requires no prophetic vision to foresee that it is destined to maintain its place, for a long time to come, in the affection and esteem of the profession; enriched, as it doubtless will be, in each succeeding edition, by new matter and by such changes in its arrangements as the exigencies of the science may demand. The work of Malgaigne, even if the second portion were as well translated as the first, would not be able to compete with it, as it is deficient in those references to, and details of, American practice which form such striking features in the treatise of Dr. Hamilton, who has spared no pains to render his labor exhaustive, gleaning, like an ant, his materials from every accessible source.

Dr. Hamilton divides his work into two parts, the first being occupied with fractures, and the second with dislocations. Thirty-five chapters, running through four hundred and eighty-four pages, are devoted to the first of these topics, and twenty-six to the latter. Two hundred and eighty-nine well-executed wood-cuts, many of them original, are interspersed through the text, and add greatly to the merit and value of the work. The distinguished publishers have performed their part of the enterprise with their accustomed taste and liberality, and the mechanical execution of the work, considered as a whole, will compare favorably with the best productions of the British press of the present day. Finally, the work is appropriately inscribed to Dr. Mott, as a testimony of esteem on the part of the author for a man who has done so much to ennoble American surgery, and for whom we all cherish such profound respect and veneration.

It is not our intention to present anything like a formal analysis of this work; to do so, would carry us far beyond the limits which we have assigned to us, to say nothing of the fact that it would be a matter of supererogation, inasmuch as no intelligent practitioner will be likely to be without a copy of it for ready use. No library, however extensive, will be complete without it. We shall, therefore, content ourselves with some passing comments upon such topics as may impress us particularly either on account of their extraordinary force and value, or on account of their novelty, or supposed imperfections.

The author retains the old distinction of fractures into compound and complicated, meaning, after the fashion of other writers, that in the former there is an external wound communicating with the ends of the fragments, while in the latter there may be an additional injury, as a dislocation or a severe lesion of the soft structures. To our own mind such a division has always appeared unnecessary, and even unscientific, since the whole subject might be very appropriately discussed under the head of complicated fractures, as has been done by Malgaigne and some of our writers upon general surgery.

The manner of examining a patient supposed to be laboring under fracture, is dwelt upon by Dr. Hamilton with special emphasis. We shall extract some of his remarks upon the subject, hoping thus to impress their importance more fully upon the attention of the reader. Long experience has convinced us that there are few surgeons, however capable in other respects, who are competent to examine a broken limb in a proper and unexceptionable manner.

"I cannot dismiss this subject without calling attention to the necessity of exercising care and gentleness as well as skill in the examination of broken limbs. Nothing, in my opinion, betrays a lack of judgment as well as of common humanity on the part of the surgeon, so much as a rude and reckless handling of a limb already pricked and goaded into spasms by the sharp points of a broken bone. It is not enough to say that such rough manipulation is generally unnecessary; it is positively mischievous, provoking the muscles to more violent contractions; increasing the displacement which already exists, and not unfrequently producing a complete separation of impacted, denticulated, transverse, or partial fractures, which can never afterwards be wholly remedied; augmenting the pain and inflammation, and not unfrequently, I have no doubt, determining the occurrence of suppuration, gangrene, and death."

In speaking of the repair of broken bones, at page 47, the author advocates the idea that the ends of the fragments sometimes unite by "direct reunion" of the broken surfaces, without the intervention of any reparative material, in the same manner as the soft tissues occasionally unite. He adds that this mode of repair is not unfrequent in the spongy bones and in the articular extremities of the long bones, especially in cases of impaction, or where one fragment is forcibly driven into the other, as in certain fractures of the neck of the humerus and of the femur. For ourselves, we cannot believe in the possibility of such an occurrence. To our mind, indeed, the very conception of it is an impossibility. Whenever a broken bone is thoroughly and completely consolidated, the connecting bond must always be of an osseous nature. We might as well believe that two pieces of tin could be effectually soldered together without the agency of lead, as that a fracture could be properly repaired without the intervention of new osseous matter.

The chapter on the "General Treatment of Fractures" is replete with judicious precepts, and is worthy of attentive study. The author strongly insists upon the importance of care and gentleness in the handling and examination of a broken limb, the transportation of the patient, and the removal of the clothing, with a view to a thorough diagnosis of the case.

"Rude or awkward manipulations, by which needless pain is inflicted, are not simply acts of wanton cruelty, but they are sources, and I think I may say frequent sources, of inflammation, suppuration, and gangrene. Here, as in all the subsequent handlings, everything should be done slowly, thoughtfully, and systematically. Yet it is difficult to state the precise manner in which the surgeon ought to proceed. Much will depend upon the circumstances of the case, something upon one's natural tact, and upon the amount of experience, but more, I think, upon natural kindness of heart, and social education. The man of refinement and sensibility will know instinctively how to proceed, and needs no instruction. They who lack these qualities can never learn, and it would be quite useless to undertake to teach them. I sincerely wish such men as these latter would find some more suitable employment than the practice of a humane art."

We are glad to find in Dr. Hamilton so decided an advocate for the early coaptation of fracture. He declares that broken bones ought, as a general rule, liable to few exceptions, to be restored to their natural relations within as short a period as possible after their occurrence; a precept in which, we are quite sure, every judicious surgeon will fully concur. We do not find any allusion, in the otherwise excellent directions of the author, to the use of anæsthetics in the examination and adjustment of fractures in persons of a nervous, excitable temperament, or where the parts, in consequence of the violence of the injury to which they were subjected at the moment of the accident, have become exquisitely tender and sensitive, so as to render manipulation a source of great suffering. We have ourselves derived great assistance from the administration of ether and chloroform under such circumstances, and we can therefore strongly recommend the practice to the consideration of our readers.

The author does not seem to entertain a very high estimate of the immovable apparatus in the treatment of fractures, and his remarks upon the subject are, perhaps, in the main quite correct. Its employment certainly demands much more care and judgment than fall to the lot of most private practitioners. In the hospital, where a constant surveillance may be exercised, it is less objectionable, but even here it should not be resorted to without due deliberation. We are ourselves an advocate of its use under certain restrictions, one of the principal and most cogent of which is all absence of inflammatory excitement and swelling in the soft parts. Hence we are opposed to the practice of Mr. Erichsen and other surgeons, who have recourse to the treatment a few hours or days after the occurrence of the injury. The following extract will serve to show the author's views upon the subject under discussion:—

"The cases, then, to which this apparatus seems to be adapted are: a few examples of transverse or serrated fractures in which the bones have not become displaced, and in which little or no swelling is anticipated; and in certain fractures which were originally more complicated, but in which a partial union, and the subsidence of the inflammation, have reduced them to a more simple condition; and especially in cases of delayed union. If now the dressings are applied carefully, the bandage being only moderately tight, and a portion of the extremity of the limb is left uncovered so that we may observe constantly its condition, and at proper intervals the apparatus is opened completely in order that we may subject the whole limb to a thorough examination; in such cases as we have now indicated and with such precautions, we admit that the 'apparatus immobile' constitutes an invaluable surgical appliance, and one of which no surgeon can well afford to be deprived."

Dr. Hamilton speaks in a condemnatory manner of the practice of treating fractures simply with the roller, without the aid of splints and extending apparatus, originated by Radley, of England, and so constantly and warmly advocated by Dr. Dudley, of Kentucky, pronouncing it as irrational, and as, in his opinion, unworthy of confidence. He regards as still more unscientific, and even absurd, the practice of Jobert, of Paris, who discards both bandages and splints in the treatment of all, or nearly all, fractures of the long bones, using merely extension to maintain apposition of the ends of the fragments. To these sentiments we most cordially subscribe, satisfied that the practice here referred to is not only irrational, but wholly unwarrantable. The "Dudley treatment," as it was wont to be called in the West and Southwest, has been productive of immense mischief, having been a cause not only of frequent deformity, but also of loss of limb, and of not a few suits for malpractice.

The following paragraph relates to an important subject, not perhaps sufficiently understood and acted upon by practitioners:—

"The rule generally laid down by surgeons, that we should at once close the wound in compound fractures, with sutures and adhesive straps if necessary, or with bandages, is far too absolute. This practice will do when there is no great contusion or extravasation of blood, but if blood is flowing, it is much better to leave the wound open so as to permit it to escape freely; and if the severity of the injury warrants the supposition that much inflammation is to ensue, the danger of gangrene is greatly lessened by thus allowing the opening to remain as a channel of exit for the inflammatory effusions."

The subjects of "Delayed and Non-union of Broken Bones" are well discussed, the author having freely availed himself, in this portion of the work, of the monograph of Dr. Norris, of this city, "On the Occurrence of Non-union after Fractures," which he justly considers as the most complete and reliable in any language. He agrees with Walker, Lonsdale, Liston, Hammick, Malgaigne, and others, in regard to the great rarity of this accident, and states that in his own practice he has noticed it much oftener in the humerus than in the femur, which was the bone most frequently involved, in the statistics of the Philadelphia surgeon. We have ourselves seen this non-union in nearly equal proportion in both of these pieces.

The treatment of ununited fracture has engaged a great deal of attention, and has been a prolific source of discussion and controversy. Physick, as is well known, was partial to the seton, which he was the first to employ in a case of broken humerus early in the present century, although it had already been suggested by Winslow, in 1787. The author, in his summary of the treatment of this accident, speaks of it as an "extreme measure." "If," says he, "the fracture is not in the femur, and as an extreme measure, employ the seton." We do not, we confess, perceive any necessity for such excessive precaution. If properly used, we believe that the seton is one of the most harmless, as it is certainly one of the most efficient, modes of treatment that can be adopted in ununited fractures, no matter where situated. It is easy to perceive that mischief may follow its injudicious application, and we have got the statistics of Dr. Norris to show that its employment has occasionally been attended by violent inflammation, extensive suppuration, erysipelas, and even death; but such accidents will rarely, if ever, happen, if proper attention be paid to the state of the system prior to the introduction of the seton, and, above all, if care be taken not to let it be retained in the parts beyond a few days.

Dr. Hamilton is of opinion that resection is applicable only to superficial bones, and in cases of overlapping. We would say emphatically that the operation is applicable to all cases of ununited fractures, whatever bones they may implicate. When the ends of the fragments have become much accuminated from absorption, or when they are connected by dense fibrous tissue or by a false joint, especially when all the more ordinary means have failed to afford any benefit,—the practitioner has no alternative; he must resect or amputate, or abandon the case as hopeless. Who would hesitate about a choice?

In delayed union, or, more properly speaking, retarded consolidation, the surgeon has at his command a great variety of measures, all, if aided by appropriate constitutional treatment, more or less efficient. Among these are friction of the ends of the fragments, acupuncture, compression, blisters, tincture of iodine, electricity, the insertion of ivory pegs, as advised by Dieffenbach, subcutaneous puncture, and perforation of the extremities of the broken pieces—an operation first suggested by Dr. Brainard, of Chicago. These methods of treatment are easy of execution, free from danger, and, either singly or combinedly, generally adequate to a cure, although much perseverance is often necessary in their use. In cases, on the contrary, of long standing, particularly where the ends of the fragments are incrusted with adventitious matter, excessively indurated, or sharp and rounded, little if any benefit is to be expected from their employment. We do not except from this remark the vaunted plan of Dr. Brainard; for our own experience, and a careful examination of the published cases of that gentleman, said to have been treated in this way, convince us that it is only in cases of retarded reunion that it is entitled to any of the credit that has been claimed for it by its author.

The chapter on "Bending and Partial Fractures of the Bones" is one of the most interesting and valuable in the book, and deserves an attentive perusal. It is interspersed with an account of the author's own experiments and observations upon these affections; a circumstance which not a little enhances the reader's confidence in his opinions and practice. If space permitted, we should gladly transfer some of his remarks and conclusions.

It would occupy too much of our time to examine, with a view to just criticism, all that Dr. Hamilton has written upon fractures of particular bones; we have bestowed upon this portion of the work an attentive perusal, and have risen from our task with the conviction that nearly all these topics have been treated with consummate ability, every chapter and section being characterized by great diagnostic tact and sound practical sense.

Under the head of "Fractures of the Shaft of the Femur," the author introduces the subject of adhesive plaster bands as means of making permanent extension, declaring that they deserve a decided preference over all other methods at present known to surgeons; an opinion in which every practitioner of experience will heartily concur. We have ourselves long employed this method, and can therefore speak most unequivocally in its favor.

Dr. Hamilton appears to be in doubt as to the inventor of this plan of treatment. A thorough investigation of the subject leaves no doubt in our own mind that it was first described by Dr. Gross, in his work on the "Diseases of the Bones and Joints," published in this city in 1830. From the subjoined extract from that work, it will be perceived that he speaks of it as having first witnessed it in the practice of his early preceptor, Dr. Swift, of Easton, Pennsylvania.

"In complicated fractures of the leg, it not unfrequently happens that the soft parts about the ankle are so much contused, or otherwise injured, as to render it impossible to employ the usual extending bands. When this is found to be the case, the difficulty may usually be remedied by applying along each side of the leg, as high up as the seat of the fracture will admit, a piece of strong muslin about two feet and a half in length, two inches and a half in width, and spread at one of its extremities with adhesive plaster. The part which is applied upon the limb should be confined by three or four circular strips, so as to keep it firmly in its place, and equalize the extending power. The free extremities of the extending bands should then be tied under the sole of the foot, and be secured to the block or bar which connects the lower ends of the splints. This mode of making extension, for which we are indebted to the ingenuity of my friend and preceptor, Dr. Swift, of this place, will, I am fully persuaded, be found highly useful in practice, and satisfactorily obviate the inconveniences to which I have just alluded."

It may be added that the work of Dr. Gross here referred to, although published within less than three years after he commenced the practice of medicine and surgery, must have been well known to the American profession, as it was used as a text-book upon diseases of the bones and joints in some of our schools, especially in some of those in New England, and we may therefore legitimately conclude that the above extract must have attracted more or less attention on the part of our surgeons; a circumstance which probably accounts for some of the claims that have since been set up in other quarters. We may also add, that in a conversation which we have recently had with an eminent practitioner of this city, Dr. Samuel Jackson, formerly of Northumberland, he informed us that he had resorted to the use of adhesive straps as a means of extension, in a case of compound fracture of the leg, as early as 1814. He, however, never published anything upon the subject, and the consequence was that the attention of the profession was not called to it until after the appearance of the work of Dr. Gross.

Twenty-two pages or more are devoted to the prognosis of intra-capsular fracture of the femur, or, in other words, to the adjudication of the question of the possibility of osseous union in this variety of accident—the author passing in review, with much caution and apparent impartiality, the most prominent facts that have been published upon this interesting and still vexed subject, both at home and abroad; and he finally comes to the conclusion that, although such an event is possible, yet he has himself never seen a specimen which, upon a thorough examination, unequivocally established its existence. He asserts that the various preparations that have been exhibited in Europe and America, as supposed illustrations of intra-capsular fractures, were either no fractures at all, or that if they were, they extended at the same time into the bone beyond its capsule; or, finally, that they were merely examples of morbid alterations consequent upon the effects of old age, arthritic disease, or what has been termed interstitial absorption or atrophy of the cervical portion of the bone. A careful perusal of the testimony adduced by the author satisfies us that his conclusion is perfectly legitimate; and we may add, that we have ourselves never seen anything tending to contradict it. While we believe that nothing is impossible, philosophically speaking, we assume that an osseous union of a fracture of the neck of the femur entirely within the capsular ligament is one of the rarest occurrences imaginable, and one which has certainly not yet been satisfactorily determined by anything that has been shown or written upon the subject.

Of the large number of apparatuses described by Dr. Hamilton for the treatment of fractures of the thigh, there is not one, according to our experience, that is really altogether unobjectionable. The construction of such a contrivance, one that shall thoroughly and readily fulfill all the indications presented in oblique fractures of the femur, is still a desideratum. That this is the fact, is shown by the difficulty that the practitioner experiences in keeping the ends of the broken bone in apposition, and by the circumstance that in nearly every case, no matter how skillfully and carefully treated, there is more or less shortening of the corresponding limb. If this does not teach us the imperfection of these appliances, what, in common sense, it may be asked, does?

The following quotations bear so closely upon this subject that they cannot fail to be of the deepest interest to the reader, to say nothing of the consolation which they tend to afford to those unfortunate members of the profession who are obliged to undergo the pains and penalties of suits for malpractice.

"In conclusion," says Dr. Hamilton, "I wish to say briefly, that in view of all the testimony which is now before me, I am convinced—

"First. That in the case of an oblique fracture of the shaft of the femur occurring in an adult, whose muscles are not paralyzed, but which offer the ordinary resistance to extension and counter-extension, and where the ends of the broken bone have once been completely displaced, no means have yet been devised by which an overlapping and consequent shortening of the bone can be prevented.

"Second. That in a similar fracture occurring in children, or in persons under fifteen or eighteen years of age, the bone may sometimes be made to unite with so little shortening that it cannot be detected by measurement; but whether in such cases there is in fact no shortening, since with children especially it is exceedingly difficult to measure very accurately, I cannot say.

"Third. That in transverse fractures, or oblique and denticulated, occurring in adults, and in which the broken fragments have become completely displaced, it will generally be found equally impossible to prevent shortening; because it will be found generally impossible to bring the broken ends again into such apposition as that they will rest upon and support each other.

"Fourth. That in all fractures, whether occurring in adults or in children, where the fragments have never been completely or at all displaced, constituting only a very small proportion of the whole number of these fractures, a union without shortening may always be expected.

"Fifth. That when, in consequence of displacement, an overlapping occurs, the average shortening in simple fractures, where the best appliances and the utmost skill have been employed, is about three-quarters of an inch."

Part II. of Dr. Hamilton's work treats, as already stated, of dislocations, and this important subject is handled in the same exhaustive manner as the portion relating to fractures, although the number of pages allotted to it is somewhat less. Under the head of general treatment, the author, after stating that many luxations may be promptly reduced by manipulation alone, points with great emphasis to the indispensable necessity of a thorough knowledge of the anatomy of the joints, or of the nature of the muscles and ligaments which antagonize the efforts of the surgeon in his attempts at restoration. His remarks upon this subject are so judicious that we cannot avoid giving them in the form of an extract.

"We must understand not only what muscles and ligaments antagonize the reduction, if we would be most successful, but also what muscles, by being provoked to contraction, will themselves aid in the reduction. In short, to become expert bone-setters in the department of dislocations, one must possess a complete knowledge of the physiognomy or the external aspect of joints, acquired only by repeated and careful examinations, he must be familiar with the anatomy and functions of the muscles, he must understand thoroughly the ligaments, he must have experience, tact, and fertility of resource.

"Without these qualifications he will do better never to undertake to treat dislocations, since he is constantly liable to mistake fractures for dislocations, and dislocations for fractures; he will submit a sprained wrist to violent extensions, under the conviction that the joint is displaced; he will mistake natural projections for deformities, and fail to recognize the real deformity when it actually exists; he will leave bones unreduced, fully believing that they are reduced; and he will all in all, within a few years, accomplish vastly more evil than he can ever do good. Let a man practice any other branch of surgery if he will, without experience or scientific knowledge, but he must not attempt to reduce dislocated bones. The most learned and the most skillful we shall find falling into error, embarrassed by the uncertainty of the diagnosis, or successfully resisted by the power of the opposing agents; what then can be expected of those who are both ignorant and inexperienced, but failures and disasters?"

In regard to Jarvis's adjuster, the author thinks it possesses some advantages over the pulleys; a circumstance which, he adds, may perhaps entitle it, at times, to a preference in certain cases. With this conclusion the result of our experience is totally at variance. We have seen a good deal of the workings of the adjuster, both in our own practice and in that of others, and we have never met with an instance where, conscientiously speaking, we thought we had derived any decided advantage from its employment. Unless judiciously managed, we consider it a dangerous apparatus, and no one will deny that it is not a most complex and awkward one.

We are somewhat surprised that the author has not been more definite, under the head of "General Treatment," on the subject of reducing dislocations by manipulation. It is true, he speaks in detail of this method afterwards, in the chapter on luxations of the hip-joint; but it seems to us that it would have been well to have discussed it here, and to have made an attempt to generalize it, or to have laid down rules for the guidance of the surgeon in the management of dislocations of the various articulations of the body. No one, we are sure, is better qualified for such a task than the learned author of this work. In the historical sketch of the reduction of luxations by manipulation, in the chapter above referred to, Dr. Hamilton adduces a great number of authorities, clearly showing that this mode of treatment dates back to the earliest ages of the profession, and that it was repeatedly employed by English and even American surgeons during the last and present centuries. Allusion is made to Dr. Reid, of Rochester, in connection with the subject; but not, as it seems to us, with that degree of prominence which that gentleman deserves for the agency which he exercised in methodizing the process, and in thus placing it conspicuously before his professional brethren. To him, more than to all other writers, is unquestionably due the merit of its general introduction.

Dr. Hamilton has added a large and interesting chapter on "Congenital Dislocations;" and although these occurrences are, in the main, extremely infrequent, yet a knowledge of them is, practically, of the greatest value, as their early detection and treatment are generally of paramount importance to a successful issue.

Finally, the work is furnished with two excellent indexes, greatly facilitating reference, and thus essentially enhancing its value.

Our task, as reviewer of this work, has been altogether a most agreeable one. We rise from it with the conviction that we have spent our time in pleasant company, and that we have profited by our labors. The book possesses great and intrinsic vitality, and will have the effect of placing its author among the foremost of those who have contributed so much of late years in enriching the medical literature of our country. The style of Dr. Hamilton is clear and vigorous, but not always free from error. Now and then we meet with an imperfect sentence, wanting an important member, marring its force and beauty. All this, however, will doubtless be corrected in another edition.

